# Sparse representation of brain signals offers effective computation of cortico-muscular coupling value to predict the task-related and non-task sEMG channels: A joint hdEEG-sEMG study

**DOI:** 10.1371/journal.pone.0270757

**Published:** 2022-07-01

**Authors:** Ahmadreza Keihani, Amin Mohammad Mohammadi, Hengameh Marzbani, Shahriar Nafissi, Mohsen Reza Haidari, Amir Homayoun Jafari

**Affiliations:** 1 Department of Medical Physics and Biomedical Engineering, School of Medicine, Tehran University of Medical Sciences, Tehran, I.R. Iran; 2 Research Center for Biomedical Technologies and Robotics (RCBTR), Tehran University of Medical Sciences, Tehran, I.R. Iran; 3 Department of Electrical and Computer Engineering, University of Tehran, Tehran, I.R. Iran; 4 Department of Biomedical Engineering, Amirkabir University of Technology (Tehran Polytechnic), Tehran, I.R. Iran; 5 Department of Neurology, Neuromuscular Research Center, Shariati Hospital, Tehran University of Medical Sciences, Tehran, I.R. Iran; 6 Section of Neuroscience, Department of Neurology, Faculty of Medicine, Baqiyatallah University of Medical Sciences, Tehran, I.R. Iran; Swansea University, UNITED KINGDOM

## Abstract

Cortico-muscular interactions play important role in sensorimotor control during motor task and are commonly studied by cortico-muscular coherence (CMC) method using joint electroencephalogram-surface electromyogram (EEG-sEMG) signals. As noise and time delay between the two signals weaken the CMC value, coupling difference between non-task sEMG channels is often undetectable. We used sparse representation of EEG channels to compute CMC and detect coupling for task-related and non-task sEMG signals. High-density joint EEG-sEMG (53 EEG channels, 4 sEMG bipolar channels) signals were acquired from 15 subjects (30.26 ± 4.96 years) during four specific hand and foot contraction tasks (2 dynamic and 2 static contraction). Sparse representations method was applied to detect projection of EEG signals on each sEMG channel. Bayesian optimization was employed to select best-fitted method with tuned hyperparameters on the input feeding data while using 80% data as the train set and 20% as test set. K-fold (K = 5) cross-validation method was used for evaluation of trained model. Two models were trained separately, one for CMC data and the other from sparse representation of EEG channels on each sEMG channel. Sensitivity, specificity, and accuracy criteria were obtained for test dataset to evaluate the performance of task-related and non-task sEMG channels detection. Coupling values were significantly different between grand average of task-related compared to the non-task sEMG channels (Z = -6.33, p< 0.001, task-related median = 2.011, non-task median = 0.112). Strong coupling index was found even in single trial analysis. Sparse representation approach (best fitted model: SVM, Accuracy = 88.12%, Sensitivity = 83.85%, Specificity = 92.45%) outperformed CMC method (best fitted model: KNN, Accuracy = 50.83%, Sensitivity = 52.17%, Specificity = 49.47%). Sparse representation approach offers high performance to detect CMC for discerning the EMG channels involved in the contraction tasks and non-tasks.

## Introduction

Functional cortico-muscular coupling (FCMC) in different rhythms plays the main role in sensorimotor control [1]. This index could be utilized to indicate the interaction between the brain motor cortex and associated body muscles [2]. Conventionally, the common method that describes this connection is cortico-muscular coherence (CMC) analysis [2]. CMC has been shown noninvasively and invasively with MEG/EEG and LFP signals in humans and monkeys, respectively [3–5].

Extensive studies have reported the coupling/synchronization of alpha-band (8-14Hz) during static/isometric contractions, finger movements and the transition between force targets [6–8]. On the other hand, beta rhythm (15–30) coupling has been indicated as the main contributor to movement control [9, 10]. Maximum beta rhythm coupling is observed during maintaining steady-state force and also controlling the fine motor control tasks [5, 11]. Other studies have reported the role of gamma rhythm oscillation (30–80 Hz) in producing the strong and dynamic forces [5, 8, 12, 13].

These rhythms define the different roles of each neural oscillation communication /interaction between the central nervous system and the peripheral muscles [2]. A variety of parameters such as demographic data, task data, frequency range of rhythm, etc. influence these rhythms and have an effect on the CMC value [2].

Although CMC has been studied in an extensive body of research for healthy subjects and sports-related disorders, the objective of its applications is still ambiguous as the value of CMC varies among individuals, and so the detection of this index is still challenging.

Overall, CMC analysis which detects the presence of synchronization in the EEG-EMG recordings from the brain and concurrently active muscles, is one of the most common signal processing methods [2, 12]. However, some factors can affect the magnitude of CMC and therefore synchrony between the EEG-sEMG signals becomes difficult to detect. One factor is the time delay between the synchronized events in the brain and the muscles which can be described as misalignment bias [14]. However, the main cause of low-level synchronization between EEG-sEMG signals is the presence of non-task-related noise during motor/contraction tasks [15]. Different approaches have been proposed toward increasing the FCMC while most of the studies have focused more on the single projection of each EEG channel on the sEMG channel [2, 14, 16–18]. Several studies have indicated the role of multichannel projection of the EEG signals on sEMG channel and found the effectiveness of this assumption on increasing the FCMC [10, 15, 19]. Denoising method based on the sparse signal representation via blind source separation has been shown to increase CMC value [15]. However, here, we aim to use the assumption of sparse representation with different points of view and also to solve a different problem i.e., to detect the task-related and non-task sEMG channels via spatially sparse projection of high-density EEG (hdEEG) on each sEMG data during four different contraction tasks. In summary, we hypothesize that the sparse contribution information of EEG channels during the contraction tasks could effectively calculate the cortico-muscular value for prediction of the task-related and non-task sEMG channels, and also that these sparse coefficients could be included as coefficients of a spatial filter in relevant studies.

## Material and methods

### Participants

Fifteen healthy normal subjects (30.26 ± 4.96 years, 8(M)) were enrolled in the study. Subjects reported no history of neurologic and psychiatric disorders and head trauma. They participated via an announcement through social media. Before the study, each subject was informed about the project and signed an informed consent form based on the Declaration of Helsinki. The study was approved by the Medical Research Ethics Committee of the Tehran University of Medical Sciences with code number IR.TUMS.MEDICINE.REC.1398.459 on September 2, 2019. The individuals in this manuscript have given written informed consent (as outlined in PLOS consent form) to publish these case details (i.e., images in Fig 2 and short video clips 1–4 as supporting information). Demographic data of the participants are presented in Table 1.

**Table 1:** Demographics and order of tasks for the subjects enrolled in the study.

Subject’s Code	Age (mean = 30.26, SD = 4.96)	Height (mean = 171.6, SD = 8.52)	Weight (mean = 68.66, SD = 14.19)	Gender (M:8, F:7)	Task order (Random)
**S1**	32	164	53	F	1-2-3-4
**S2**	28	175	75	M	2-4-1-3
**S3**	22	175	81	M	2-1-4-3
**S4**	29	173	59	F	3-4-2-1
**S5**	25	170	62	M	3-1-2-4
**S6**	27	167	70	F	3-2-4-1
**S7**	36	185	88	M	2-4-1-3
**S8**	31	185	85	M	1-3-2-4
**S9**	30	180	80	M	2-1-4-3
**S10**	32	176	78	M	3-2-4-1
**S11**	36	160	51	F	2-4-1-3
**S12**	39	165	60	F	1-2-3-4
**S13**	27	167	52	F	3-2-4-1
**S14**	24	156	49	F	2-1-3-4
**S15**	36	176	87	M	4-3-1-2

### Task design and experimental setup

Four different contraction tasks were completed by each subject in random order:

Task 1: A static task in which the subjects tried to hold the force output between 15% -45% of maximum volume contraction (MVC) by pressuring a force sensor with hand middle finger.

Task 2: A dynamic contraction task in which the subject closed fist and opened hand with normal voluntary speed based on the task timeline.

Task 3: A static task that subjects generated a force between 15%-45% MVC with contraction of their foot on the device that is designed like a pedal.

**Fig 1:**
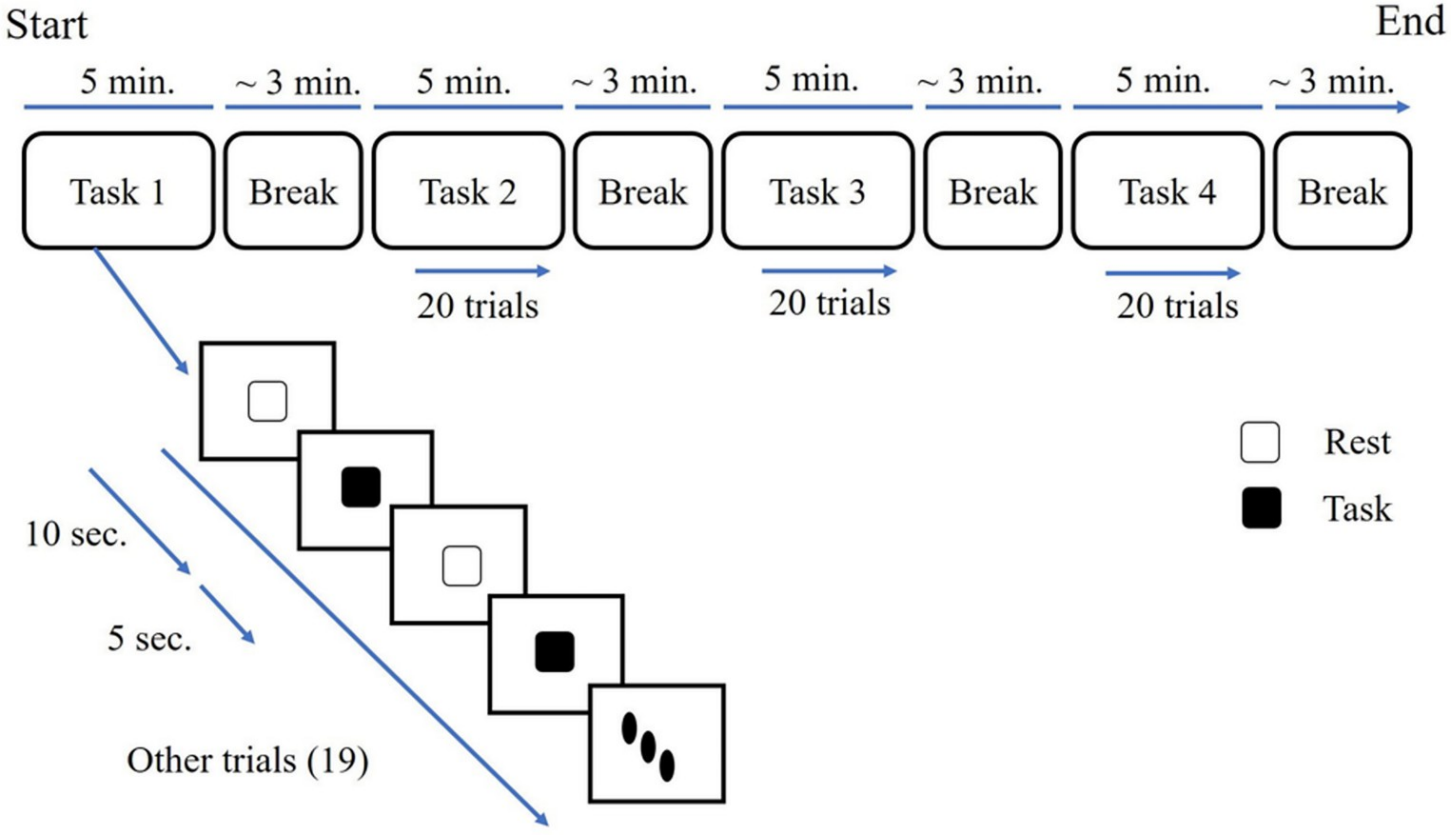
Schematic illustration of the timeline for a complete session is sketched. For instance, in this figure, we have shown a session with the task order of 1-2-3-4. Each session included 4 tasks (2 dynamic and 2 static tasks). In each task, subjects completed 20 trials. The tasks were randomly presented (Note: Here we only showed the 1-2-3-4 order. Other random sequences are presented in Table 1 which shows the specific order for each subject)). Between switching to the next task, subjects had a break time of about 3 minutes. In each trial, the contraction time was 5 seconds and the rest time was 10 seconds. Each task took about 5 minutes. Total time of complete session without considering the preparation time was about 32–35 minutes.

**Fig 2:**
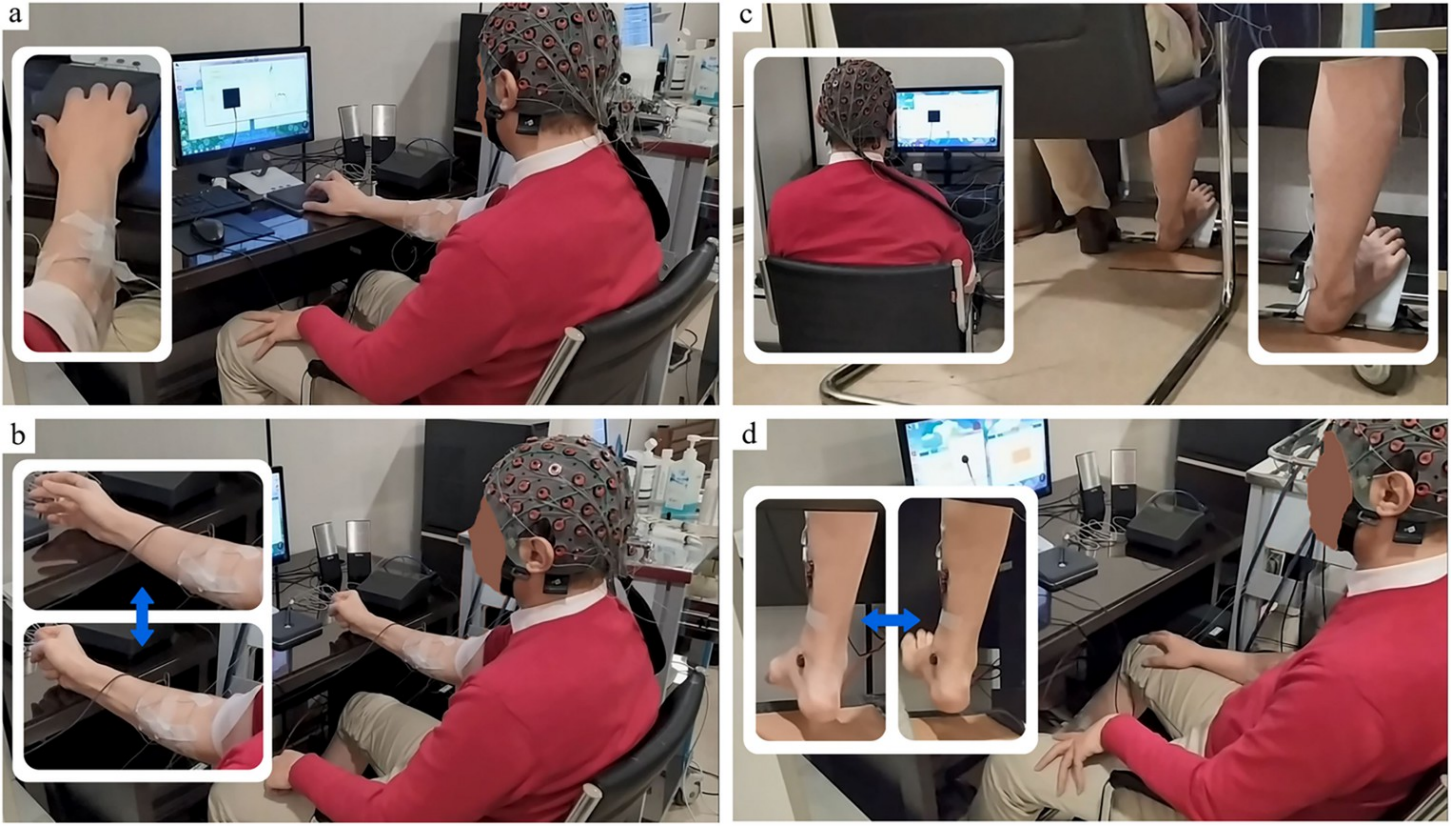
The experimental setup of the study. Four completed tasks, subjects, channels, and setup are presented in the picture. A) In task 1 as a static task; subjects did the task with the middle figure and held the output force in a specific range. B) In task 2 as a dynamic task; subjects opened and closed their hand with normal voluntary speed. C) Task 3 is static; the subjects held the pedal to generate the output force in the considered range. D) Task 4 as a dynamic task, subjects did the dorsiflexion and plantar flexion with normal voluntary speed. For all the tasks, subjects saw the monitor to do the task based on the timeline shown on screen.

Task 4: A dynamic task that the subject did the dorsiflexion-plantar flexion task according to the timeline with normal voluntary speed. Before completing the static tasks (Task 1 and Task 3) we acquired the maximum volume contraction value for each task. The subjects were asked to generate maximum force from the hand middle finger and by the soleus muscle on the designated device for contractions (Figs 1 and 2). The timeline of the task was same for all the tasks and the schematic completion of a session is shown in Fig 1.

A bi-functional device was designed to be used in static tasks. The picture of the device is presented in Fig 2. For the static finger tasks, this device could be used as 2 flat plates and the subjects could use any finger to generate output force by pressing the force sensor embedded in the device. The subjects used the middle finger for hand tasks. The device could also be used as a pedal for foot tasks and subjects put their foot on the pedal and the link between the 2 plates transformed the generated force to the sensor and subjects could see the force on the screen in front of them. We have designed a GUI that subjects could see the force and should maintain the output force between 15%-45% of their MVC. The timeline of the task was designed by a visual task with Phychotoolbox [20]. Subjects were asked to look at the monitor and when the black square is presented, they should do the task and generate suitable output force and hold it during that time. Each task was presented in 20 trials (Each trial included 10 second rest time followed by a 5-second contraction task). The trigger signals (force and psychophysics task) were sent to the acquisition computer via LTP ports of the task presentation PC. In the dynamic tasks, subjects carried out the tasks by focusing on the visual presentation of the black square shown on the screen. The force monitoring was not shown on the screen. They did the dynamic tasks with the normal voluntary speed.

Subjects sat on a comfortable chair and in front of the task presentation PC screen and g.Hiamp MultiChannel Amplifier was used for data acquisition. High-density synchronous EEG-sEMG (10/20 standard system, 53 EEG channels, 4 bipolar sEMG channels (two bipolar channels for hand and 2 bipolar channels for foot)) were acquired for each task with 1200Hz sampling frequency. The experimental setup is shown in Fig 2. Supplementary materials present short videos of how the subjects carried out the tasks (S1–S4 Videos).

Hand Extensors, Hand Flexors, Tibialis anterior (TA), and Soleus muscles were chosen based on the SENIAM protocol [21]. sEMG electrodes (Ag/AgCl electrodes) were placed on the muscle belly with 2 cm inter-electrode distance and data were gathered with bipolar measurement. An online bandpass filter (2–500 Hz) and notch filter (50 Hz) were used in the data acquisition process.

### Analytical methods, data analysis

#### Preprocessing

Preprocessing analysis of hdEEG data was based on the HAPPE pipeline [22]. Firstly, the EEG data were filtered with a bandpass filter (2–100 Hz), then the bad channels were detected and rejected. The Wavelet-enhanced thresholding (W-ICA) was performed before ICA analysis. This analysis removes several classes of artifacts, including eye and muscle-related artifacts, high-amplitude artifacts, and signal discontinuities (e.g., electrodes losing contact with the scalp). After W-ICA analysis, the EEG data were more suitable for ICA analysis with automated component rejection to detect the retained artifacts. When the components related to artifacts were rejected, the bad channels were interpolated and re-referenced to the average of channels. Details of each preprocessing step is reported in the HAPPE pipeline [22]. For each hdEEG data set, HAPPE generates a report table (with descriptive statistics and objective data metrics) to evaluate the performance of preprocessing.

sEMG data were filtered with the same type bandpass filter (5–200 Hz) [22]. We did not rectify the sEMG data in this study. After the preprocessing step, the hdEEG-sEMG data were segmented in separate trials based on the synchronous triggers data. Each task included 20 trails of synchronous hdEEG-sEMG data.

We truncated 1 second from the beginning and 1 second from the end of each trail and only used 3 seconds of each trail for our analysis.

#### CMC analysis

Coherence analysis indicates the temporal correlation between two signals/time series and measures the strength of the consistency of phase lag as a function of frequency [2]. The range of this index is in the range of 0 to1. The value of 1 shows the maximum temporal correlation and the 0 value shows no temporal correlation. Coherence was calculated by the fraction of cross-spectrum to the auto spectrum of signals as given below:

For given signals x(t) and y(t), the auto spectrum and cross-spectrum can be calculated as:

$$P_{xx}=\frac{1}{L}\sum_{i=1}^{L}X_{i}(f).X_{i}^{*}(f)$$ (1)

$$P_{yy}=\frac{1}{L}\sum_{i=1}^{L}Y_{i}(f).Y_{i}^{*}(f)$$ (2)

Where *X*_*i*_ and *Y*_*i*_ demonstrate the Fourier Transforms of the signals x and y for segment *i*, respectively. *L* defines the number of segments. The cross-spectrum between two signals was calculated as:

$$P_{xy}=\frac{1}{L}\sum_{i=1}^{L}X_{i}(f).Y_{i}^{*}(f)$$ (3)

After calculation of the above values, the magnitude-squared coherence was calculated with Eq 4:

$$C_{xy}=\frac{{\left|P_{xy}(f)\right|}^{2}}{{P_{xx}(f).P}_{yy}(f)}$$ (4)

x represents cortical activity signal (EEG channel) and y represents muscle activity (sEMG channel). *C*_*xy*_ was computed for each EEG-sEMG pair for all tasks. To use the significant CMC values in our analysis, we applied the confidence level/limit (CL) threshold as described in [23].


$$CL=1-{\left(1-\alpha \right)}^{\frac{1}{N-1}}$$ (5)

Where N is the number of data segments used for CMC calculation (80) and *α* is the level of confidence (*α* = 0.95).

The mean of significant coherence values was calculated (Eq 6) for the whole frequency range. *M*_*sCoh*_ is a vector with the magnitude of 1×*p* as the rows of the input data for machine learning algorithm in the next step to discriminate sEMG channels also contributed to detect task from non-task. Columns of *M*_*sCoh*_ are EEG channels and we considered them as the predictors in machine learning algorithms.


$$M_{{sCoh}_{p}}=\frac{1}{L}\sum_{f_{min}}^{f_{max}}C_{{xy}_{p}}-CL$$ (6)

#### Sparse analysis of synchronous hdEEG-sEMG signals

To tackle the limitations of CMC method and enhance the cortico-muscular coupling values, we employed the sparse analysis while considering the projection of all EEG channels on each sEMG data. We translated the least absolute shrinkage and selection operator (LASSO) algorithm [24] in our application to extract sparse coefficients for describing the contribution strength of EEG channels on each sEMG channel during the contraction tasks. By using sparse constraints, this method provides low variance and high interpretable solution for the cortico-muscular problem as a linear regression model [10, 14]. Therefore, by considering the projection of multichannel EEG data on each sEMG channel as a linear model in Eq 7.


y=Xβ+ε (7)

Where, *y* is an *n*×1 vector which represents each sEMG channel and *X* is an *n*×*p* matrix that contains the multi-channel EEG signals. *p* indicates the number of EEG channels (53) and *n* is the length of the synchronous trials used in the analysis. The length of joint EEG-sEMG signals is equal and presented by *n*.

The LASSO algorithm solves the below optimization problem to find the sparse coefficients $\tilde{\beta }$. These coefficients demonstrate the strength of each EEG in the relevant sEMG channel.


$$\tilde{\beta }=arg\;min({\left\|y-X\beta \right\|}_{2}^{2}+\lambda {\left\|\beta \right\|}_{1})$$ (8)

Where *λ* is a penalty parameter to help achieve sparse solution for $\tilde{\beta }$. ‖.‖_2_ *and* ‖.‖_1_ indicate *l*_2_ and *l*_1_ norms, respectively. The contribution of each EEG channel was calculated from the calculated $\tilde{\beta }$ coefficients as below:

$${CD}_{p}=\left\{ \begin{array}{c} {\tilde{\beta }}_{p}\;if\;{\tilde{\beta }}_{p}>0\\ 0\;else \end{array}\right.$$ (9)

**Fig 3:**
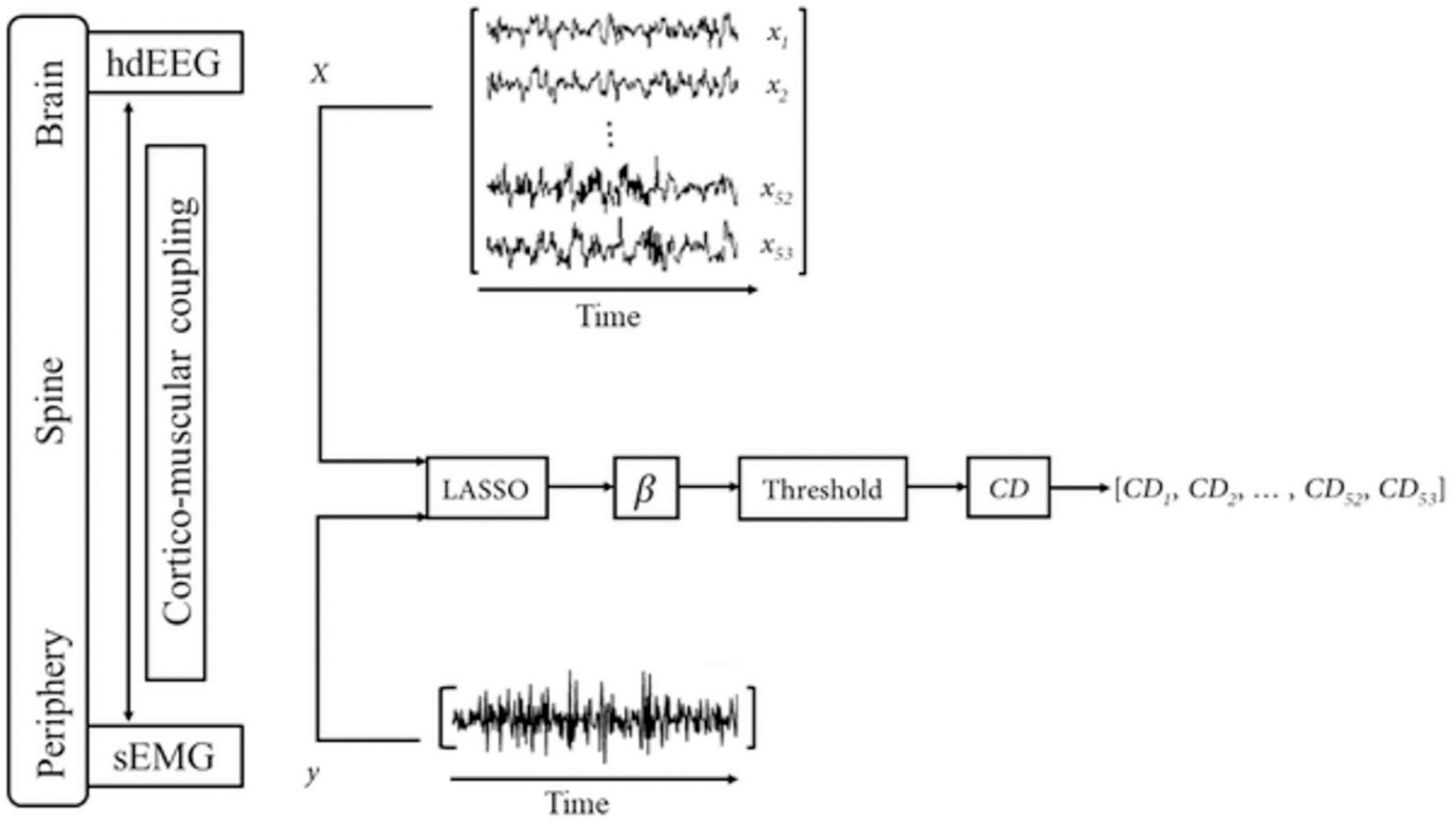
General block diagram of application of sparse analysis (i.e., LASSO method) and extracted coefficients. We schematically tried to show how the multichannel hdEEG data could be projected on each sEMG channel on the right side of the figure. The final *CD*_*i*_ coefficients for all EEG channels are utilized as input data for machine learning method. These coefficients demonstrate the coupling values and the contribution of each respective channel in the task.

The contribution degree (CD) for each trial is a vector with the magnitude of 1×*p*. These vectors construct the rows of the input table and EEG channels were considered as predictors in columns of the input table for machine learning algorithm. The general process of translating the LASSO algorithm to our problem and calculating the CD for each trial is sketched in Fig 3.

#### Table of predictors

For each method, table of data was constructed separately. In both analyses, we had a vector with 1×53 for each trial. Each column of data defined the coupling value for each EEG channel. The analysis was run on single trials. 80% of data were used for the training step and 20% was allocated for the test step. In reporting each subject’s classification result we used leave one out method. It means that for each subject, all the trials from 14 subjects were used for training step and the trials of one remained subject were used for test analysis. The accuracy, sensitivity, and specificity measures were used as evaluation criteria. Each analysis was performed for every task and a general model was also created by considering the whole task-related and non-task trials at the end.


$$Sensitivity=\frac{TP}{TP+FN}$$ (10)

$$Specificity=\frac{TN}{TN+FP}$$ (11)

$$Accuracy=\frac{TN+TP}{TN+FP+TP+FN}$$ (12)

Where;

True Positive (TP): Task-related sEMG channel correctly classified as task-related.

True Negative (TN): Non-task sEMG channel correctly classified non-task.

False Positive (FP): Task-related sEMG channel wrongly classified as non-task.

False Negative (FN): Non-task sEMG channel wrongly classified as task-related.

#### Optimized machine learning approach

Machine learning approach commonly transforms the problem into an optimization problem to be solved normally. Multiple hyperparameters have to be set before training process and defined as to how best the model fits the data [25]. Manual and automatic search are two types of parameter selection. Manual search is hard to be applied by non-experts. Therefore, automatic methods (i.e., grid and random searches) have been proposed [26]. Selecting the best-fitted model with tuned hyperparameters is an optimization problem with the black-box objective function. Thus, common optimization solutions like Newton or gradient descent could not be employed.

Studies propose Bayesian optimization for these kinds of problems as this method outperforms other global optimization methods [25, 27, 28]. Bayesian optimization solves a function that doesn’t have a closed-form. This algorithm comprises of 2 principal steps (Eq 13 and Eq 14) where DATA1:t−1={xn,yn}t−1n=1 defines the training dataset with the *t-1* observation of unknown function.

Bayesian optimization workflow for training dataset:

1. For *t = 1*,*2*, *…*
2. Maximize acquisition function (*a*) over f and finding the new point as *x*_*t*_
$$x_{t}=argmax\;a(x|{DATA}_{1:t-1})$$ (13)
3. Posterior distribution update
$$y_{t}=f(x_{t})$$
$${DATA}_{1:t}=\{{DATA}_{1:t-1},(x_{t},y_{t})\}$$ (14)
4. End For.

Statistics and Machine Learning Toolbox™ (MATLAB and Release 2020b, The MathWorks, Inc., Natick, Massachusetts, United States) was used for automatic machine learning algorithm selection with tuned hyperparameters. The optimization algorithm is already implemented in the machine learning toolbox of MATLAB software and can be used by employing the “fitcauto” function [29–31].

The Bayesian optimization, in general, finds a point where the objective function has extremum. Using the context of the “fitcauto” method, a point is a learner type alongside hyperparameters of the learner. The objective function is the cross-validated classification error. The Bayesian optimization method in “fitcauto” includes a multi-TreeBagger model of the objective function (the objective function of this model differs from the Gaussian process model implemented by other machine learning toolbox functions that mainly use Bayesian optimization). Here, an acquisition function (i.e., expected improvement) determines the next point to be evaluated. “fitcauto” method chooses the point with the minimum objective function value from among the points evaluated during the optimization as output. This method selects between the most applicable machine learning methods (e.g., ensemble classification model, k-nearest neighbor model, support vector machine classifier (SVM), discriminant analysis classifier, linear classification model, Naive Bayes classifier, and binary decision classification tree) and automatically finds the best method with tuned hyperparameters for training data. Once the optimization process is completed the “fitcauto” returns the trained model on the entire train data set, which is expected to best classify new data. For more detail on implementing the mentioned method, see Mathworks [32].

#### Statistical analysis

Statistical analyses were performed using SPSS (IBM Corp. Released 2011. IBM SPSS Statistics for Windows, Version 20.0.) to evaluate the grand average coupling values for both CMC and sparse analyses. Our analysis was run on the same group of subjects for task-related and non-task conditions. Appropriate nonparametric analysis (i.e., Wilcoxon Signed-Rank Test) was chosen for paired wise comparisons because of the data failing to provide criteria for normal distribution. The significance level α = 0.05 was employed to overall demonstrate any significant changes between the task-related and the non-task coupling values.

## Results

**Fig 4:**
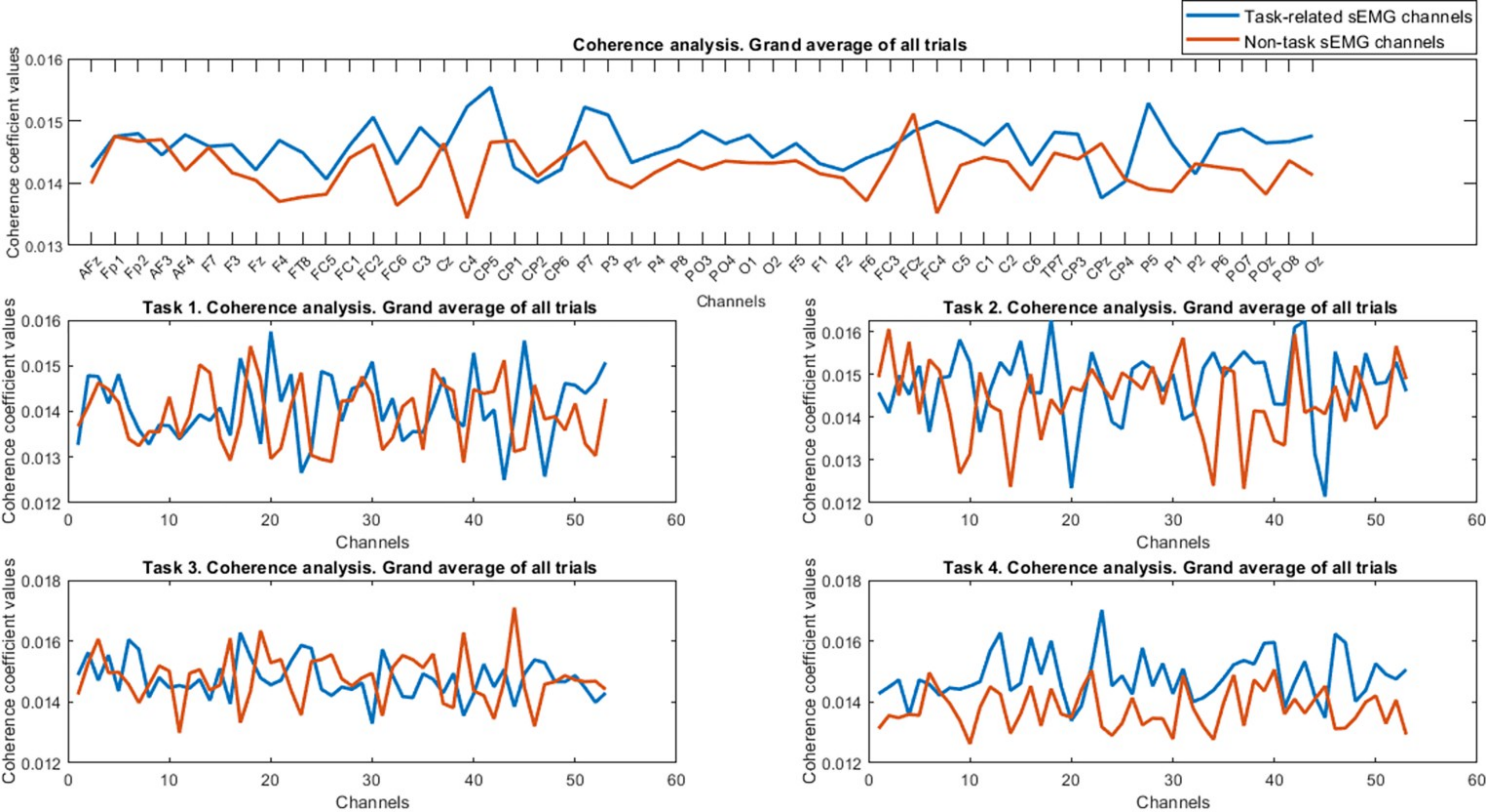
Results of coherence analysis for whole trials are depicted. Generally, we couldn’t judge whether stronger coupling values existed or not for the task-related sEMG channels compared to the non-task channels in row 1. In rows 2 and 3, the grand average results of total coherence for each task are presented. Almost higher coupling values for tasks 2 and 4, but for task 1 and task 3, the values did not follow a consistent pattern along channels and we can see higher values only for specific channels in task-related sEMG channels. The legend for all subplots is the same as row 1.

**Fig 5:**
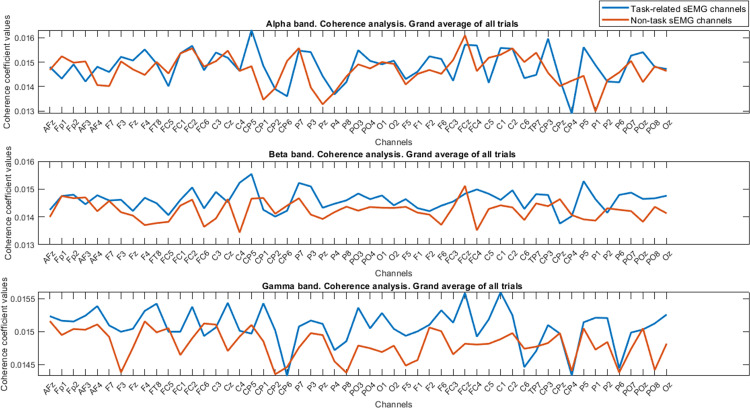
The results of coherence analysis for each specific band are described for all trials. Row 1 shows the alpha band coupling values, row 2 is dedicated to beta band coupling values, and row 3 refers to the gamma band coupling values.

**Fig 6:**
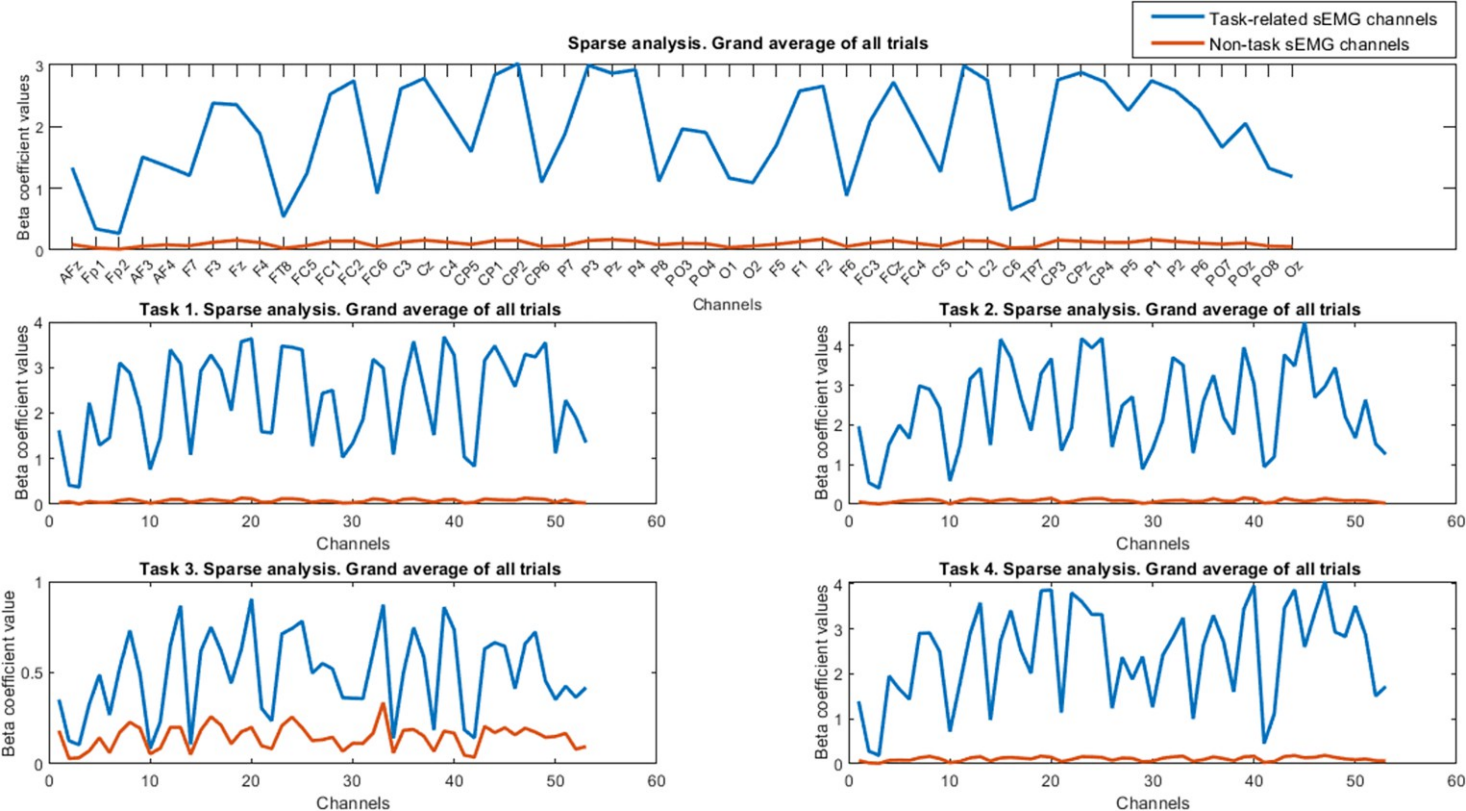
Grand average of sparse analysis results for all trials. The stronger coupling values for task-related sEMG channels are observable for all trials and also in all trials of each task. Row 1 shows the results for total trials. Rows 2 and 3, respectively show the results of all trials for each task. The legend for all subplots is the same as row 1.

In this section, firstly, the results for grand average of coupling values in both coherence and sparse analysis in each task and also for all the tasks as shown in Figs 4–6 will be presented. Then the results of applying machine learning methods for discerning the task-related sEMG channels will be mentioned (Fig 8).

As it can be observed, the grand average of the task and the non-task coupling values have different trends for coupling values along the EEG channels. However, they did not have significant differences. A Wilcoxon signed-rank test indicated that there was no significant change between these two conditions for total coherence (Z = -0.925, p = 0.355, task-related median = 0.0149, non-task median = 0.0150). Therefore, the total coherence coupling values for the task-related sEMG channels were not higher than the non-related channels.

After obtaining the general coherence index, we also calculated the grand average of coherence index for all the task and non-task-related trials in each specific frequency band (e.g., alpha, beta, and gamma). The results of coupling values using coherence analysis in each band are presented in Fig 5.

A Wilcoxon signed-rank test result showed that alpha band is not significant (Z = -1.518, p = 0.129, task-related median = 0.0148, non-task median = 0.0147) while significant results were seen in both beta band (Z = -5.006, p< 0.001, task-related median = 0.0146, non-task median = 0.0142) and gamma band (Z = -5.405, p< 0.001, task-related median = 0.0150, non-task median = 0.0148). From Fig 5, it is obvious that the coherence index is higher in both the beta and gamma ranges as our statistical analysis confirmed. Furthermore, from statistical results and Fig 5, for the alpha band, we could clearly observe the increase and decrease of coherence index in the task-related channels compared to the non-task channels, respectively.

The results of the time-domain index (i.e., sparse analysis) showed the higher coupling index in both static and dynamic tasks for the task-related sEMG channels in Fig 6.

**Fig 7:**
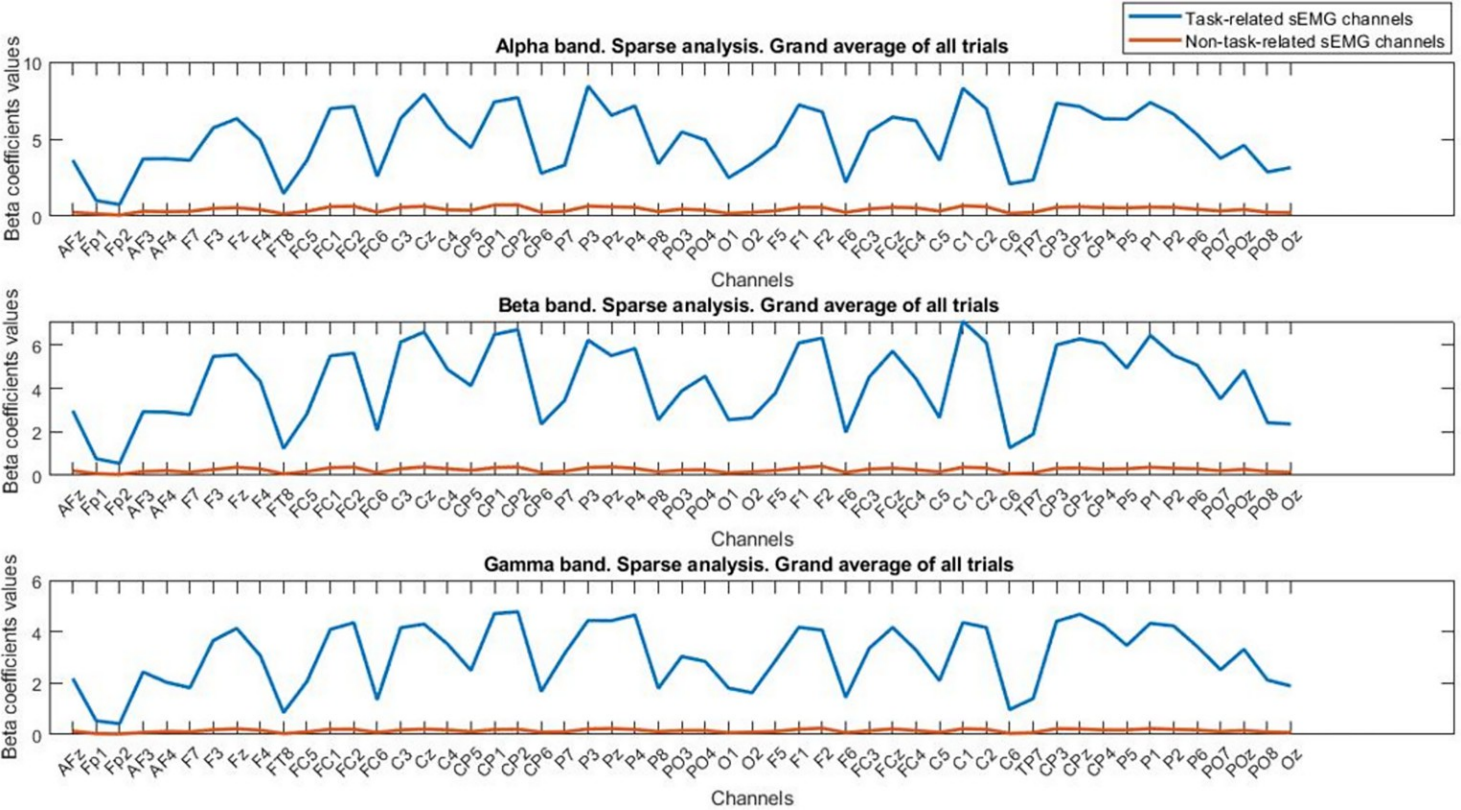
Grand average of sparse analysis results for different frequency bands. The stronger coupling values for the task-related sEMG channels are observable in all the 3 frequency ranges. The legend for all subplots is the same as row 1.

The statistical results show strong difference/significance for sparse analysis in the task-related and non-task channels (Z = -6.33, p< 0.001, task-related median = 2.011, non-task median = 0.112). The sparse analysis was also repeated for each frequency band and the relevant results are reported in Fig 7. These results confirm increase of coupling values in the task-related channel in general for four tasks. Furthermore, based on Figs 6 and 7, and statistical results, we can observe strong coupling values that were obtained from sparse analyses compared to the CMC index along the EEG channels.

In the next step, to find how much each quantitative analysis could find strong coupling values respective to the task-related sEMG channels, we analyzed these data with machine learning algorithms to objectively predict the task-related and the non-task muscle channels in single trials.

**Fig 8:**
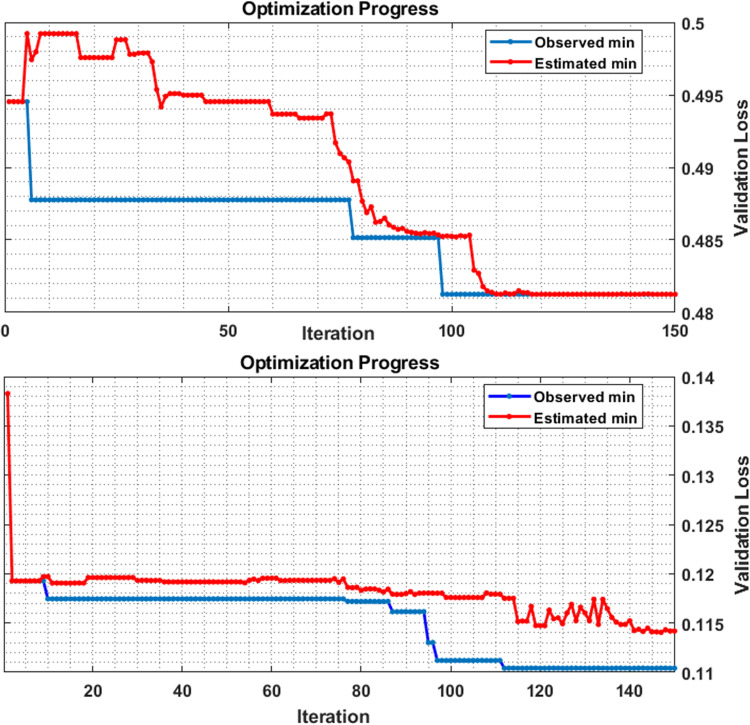
The optimization process for trained models with coherence and sparse analysis as input feeding data. Row 1 shows the training error (based on Validation Loss) for coherence coupling values on 80% of input data and row 2 shows the error of the trained model for the sparse coefficients as input data.

The validation loss for the training of machine learning method using Bayesian optimization algorithm in both the analyses is shown in Fig 8. In sparse analysis, the validation loss was lower than the coherence analysis. For better illustration of this error (Validation Loss) to compare the two methods, results of analysis of the test data are shown in Table 2.

**Table 2:** Results of application of optimized machine learning approach for the task-related and non-task-related sEMG channels.

Type of analysis	Sensitivity (%)	Specificity (%)	Accuracy (%)	Best fitted model
**Coherence analysis**	52.17	49.47	50.83	KNN
**Sparse analysis**	83.85	92.45	88.12	SVM

The maximum performance of the sparse and the coherence analysis was obtained for SVM and KNN methods respectively.

Details of the subject based classification results are reported in S1 Table and again it shows that the sparse coefficients outperform coherence coefficients for discriminating the task-related sEMG channels from the non-task-related.

This analysis was also performed again on both coherence and the sparse coefficients to classify four different contractions tasks. The results are inserted in Table 3.

**Table 3:** Results of applying optimized machine learning approach for multi-tasks classification.

Type of analysis	Sensitivity (%)	Specificity (%)	Accuracy (%)	Bets fitted model
**Coherence analysis**	46.67	55.56	51.33	Decision Tree
**Sparse analysis**	78.63	85.54	81.31	Ensemble

The result of the sparse analysis shows the efficient performance of this method in the prediction of task-related sEMG channels and also discriminating four different contractions tasks. The coherence analysis results were not suitable for detecting the task-related channels in comparison to non-task channels.

## Discussion

**Fig 9:**
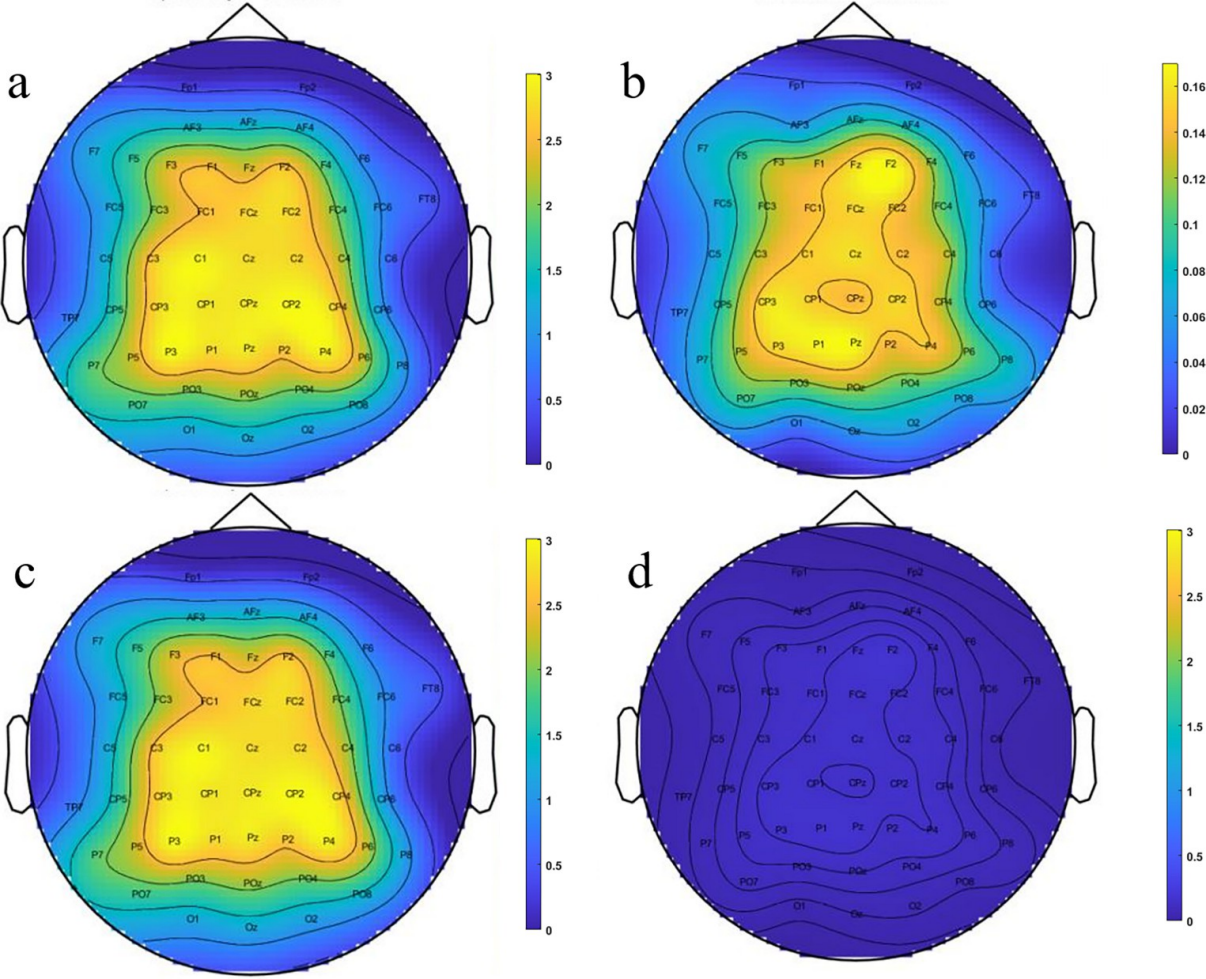
The topoplot of coupling values obtained with sparse analysis. a, b) The topoplot for the task-related (a) and non-task (b) were shown based on their specific maximum thresholds. c, d) Results after correcting the threshold for better comparison of coupling values for task-related (c) and non-task (d) channels; Both topoplots are presented based on the same threshold (i.e., the maximum threshold of both task-related and non-task values in the first row).

In this translational study, we presented utilization of sparse approach for computing the coupling between the brain signals and muscles activities. Here we have focused on the projection of high-density EEG channels on each sEMG channel to maximize the coupling index and find the task-related and non-task sEMG signals (Figs 3 and 6). Our results show that this method could help to more accurately detect the sEMG channels that are involved or have contributed in the contraction tasks (Figs 6 and 7, and Table 2). Besides, the results of applying the machine learning methods when the cortico-muscular coupling index along with the EEG channels fed as input predictors, demonstrated that the accurate performance could be achieved for discerning the task-related channels in comparison to the non-task sEMG channels and almost in the multi-tasks even in single trials ([accuracy = 88.12%, sensitivity = 83.85%, specificity = 92.45%], ([accuracy = 81.31%, sensitivity = 78.63%, specificity = 85.54%]), respectively. Most of the studies used coherence to quantify the cortico-muscular functional coupling and showed the change of this index for different motor tasks [33–37]. Our CMC results on the grand average of task-related channels showed the increase of beta and gamma band coupling values (Fig 5) [33–37]. In addition to the coherence analysis of each frequency bands, the results of sparse analysis showed increase in the coupling values for alpha, beta and gamma bands (Fig 7). These results are in the line with the previous studies, but we emphasize that the results of machine learning approach for quantifying/detecting task-related sEMG channels compared to the multi-task classification are not efficient in the single-trial coupling computation ([accuracy = 50.83%, sensitivity = 52.17%, specificity = 49.47%] vs ([accuracy = 51.33%, sensitivity = 46.67%, specificity = 55.56%]), respectively. To justify this point, we could refer to the misalignment bias and noise sensitivity of the CMC method [15]. Furthermore, the results of application of projection of multi-channel EEG on sEMG have shown that it can help in the efficient calculation of coupling index [10]. This point is important because in CMC method, only single EEG and sEMG channels contributed to calculating the CMC index [10]. In the studies that use single channels, the main drawback is that the recorded EEG channel activity represents a superposition of the signals from multiple sources of alpha, beta, and gamma oscillations, therefore not allowing to study EEG channels from other cortical areas. For instance, Bipolar and Laplacian methods work as spatial high-pass filters and partially help to alleviate the superposition problem. However, these methods do not consider information from sEMG channels, therefore, they can filter out some neuronal oscillations contributing to CMC [38–41]. Studies have suggested that using beamforming or novel multivariate regressions could be a suitable alternative [10, 42, 43]. Our approach follows this suggestion and so the sparse coefficients could be used as spatial filter coefficients. In Fig 9, we have shown a topoplot of the grand average of sparse coefficients for EEG channels that can show how each channel contributed to the task-related and non-task sEMG channels. This figure also could be an illustration of spatial filter coefficients.

There are limited studies that have shown the performance of cortico-muscular coupling in different motor tasks [33, 34, 44]. Here, we used both dynamic and static contraction tasks for both upper and lower limbs (upper limb: hand middle finger, lower limb: foot) and showed that enough strong coupling index exists in general for the four different tasks to detect the task-related and non-related muscle channels (Figs 1 and 2).

### Limitations, suggestions, applications, and future works

There are several challenges regarding the studies in the domain of cortico-muscular coupling. First of all, experimental setup and acquiring synchronous hdEEG-sEMG data needs preparation time, expertise and in this type of task, EEG data may contain more artifacts and also EMG overlap noise compares to only EEG studies. Using standard pipelines and preprocessing data is an essential point. Studies have recently proposed using a standard pipeline for the generality of EEG studies and consistent preprocessing steps for better interpretation of results in comparison to other studies. In our study, we used the HAPPE standard pipeline to quantitatively examine the quality of data and source of noises [22]. Calculating the coupling index with the rectification of EMG signals has been controversial in previous studies. We calculated the coupling values in both the coherence and sparse analysis on the non-rectified EMG channels because rectification of EMG signals may cause nonlinearity in analysis and is difficult to interpret [10]. Regarding the interpretation of CMC results, careful examination of each frequency band and the type of task is required. Several other points such as age, type of task, band frequency, filter type, window length, etc. have to be considered when comparing the results of CMC with other methods [12]. In our study, we showed that in general, the CMC value index is higher in task-related channels compared to the non-task channels in beta and gamma bands and this point is on the same page with other studies [33–37] (Figs 4 and 5). However, for neurophysiological purposes and comparison of CMC values for each specific task and band, a separate study to examine and compare each task’s results and band frequency range in depth and to compare the results with other studies is required. In our approach, we calculated the coefficients with analysis of the signals in the time domain, therefore, working in each domain needs a different interpretation from other domains. We would like to emphasize the use of multi-channel projection and time-domain features in future applications. To find the coupling for each frequency band, approach used in this study might not work perfectly. One other main challenge in cortico-muscular studies is the effect of delay between signals [14, 33]. In the current study, we did not consider the effect of delay in the analysis. In future works, it would be critical to employ the effect of delay and directionality in our analysis [45]. Recently, the studies have focused on the nonlinearity of cortico-muscular coupling and have suggested a line of research in finding a nonlinear index as a biomarker in patients in comparison to normal subjects [1, 33, 45]. In the current study, the type of method we used is linear, therefore for using these results it is recommended to consider this characteristic of approach (Fig 3). In our study, we have focused on the projection of all EEG channels on single sEMG data. One suggestion for more optimal imaging of these two sets of data may be acquiring hdEEG and hd-sEMG simultaneously and then calculating the projection of concurrent multi-channel EEG on multi-channel sEMG. This could be done in both time domain and frequency domain analyses [19].

It was difficult from the point of view of selecting the best machine learning algorithm for the problems that have no closed-form formula such as the one used in our study. We used the Bayesian optimization method to tackle this problem and selected the best-fitted model with tubed hyperparameters [28] (Fig 8).

From the perspective of the real-world application, this index could be used in rehabilitation systems for example, in stroke. We can be aware of the physiological and anatomical changes of patients based on the contraction task and the data of coupling index of EEG-EMG during the training period [33, 46, 47]. It is possible to use this index as a biomarker for early diagnosis in ALS, Parkinson’s disease and other movement disorders, brain-computer interface, robotics, etc. [12, 48, 49]. Our group is working in parallel to develop a nonlinear index based on nonlinear dynamics and chaos analysis to examine this study’s results in ALS patients compared to the normal subjects in near future. We aim to focus on nonlinearity by considering delay and directionality as these are the two important areas [33, 45].

## Conclusion

Employing sparse representation approach could provide us an effective way to compute the cortico-muscular coupling values to more accurately detect the task-related and non-task contributing sEMG channels in normal subjects. This approach outperforms the common CMC method and results have shown accurate prediction of the task-related channels for single-trial analysis using optimized machine learning approach. The strong coupling values were observed generally for the task-related channels in four different motor tasks (i.e., both dynamic and the static tasks). Based on optimization analysis for detecting the best-fitted ML algorithm with tuned hyperparameters, the SVM has the priority for the future cortico-muscular coupling studies. This index could be used in real-world applications and also the coupling coefficients could be used to construct a special filter for the EEG analysis of muscle contraction and cortico-muscular studies. There are needs for future studies to also consider the nonlinearity, delay, and directionality of the sensorimotor system for more optimal projection/imaging of brain signals (hdEEG) and muscle activities (hd-sEMG) in order to apply the results of this study on patients’ groups and compare them with the normal subjects.

## Supporting information

S1 Video Short presentation of task 1. (MP4)

S2 Video Short presentation of task 2. (MP4)

S3 Video Short presentation of task 3. (MP4)

S4 Video Short presentation of task 4. (MP4)

S1 Table Results of application of optimized machine learning approach for each subject. (DOCX)
