# Supplementary material for: Sparse representation of brain signals offers effective computation of cortico-muscular coupling value to predict the task-related and non-task sEMG channels: A joint hdEEG-sEMG study
Source: PLoS One. 2022 Jul 1;17(7):e0270757. doi: 10.1371/journal.pone.0270757 (PMC9249190; doi:10.1371/journal.pone.0270757)
Supplement: S1 Table — (DOCX) [file pone.0270757.s005.docx]

|  | Coherence analysis | | | | Sparse analysis | | | |
| --- | --- | --- | --- | --- | --- | --- | --- | --- |
| Subject's  Code | **Sensitivity (%)** | **Specificity (%)** | **Accuracy (%)** | **Best fitted model** | **Sensitivity (%)** | **Specificity (%)** | **Accuracy (%)** | **Best fitted model** |
| S1 | 43.12 | 52.50 | 47.81 | KNN | 73.12 | 98.12 | 85.62 | SVM |
| S2 | 68.75 | 30 | 49.37 | KNN | 96.87 | 88.75 | 92.81 | SVM |
| S3 | 48.75 | 45 | 46.87 | KNN | 88.12 | 86.87 | 87.50 | SVM |
| S4 | 56.25 | 48.75 | 52.50 | KNN | 98.12 | 92.50 | 93.31 | SVM |
| S5 | 43.75 | 55.62 | 49.68 | KNN | 94.37 | 86.87 | 90.62 | SVM |
| S6 | 41.87 | 51.25 | 46.56 | KNN | 85.62 | 87.50 | 86.56 | SVM |
| S7 | 53.12 | 47.50 | 50.31 | Decision Tree | 97.50 | 83.75 | 90.62 | SVM |
| S8 | 55.62 | 55.62 | 55.62 | SVM | 92.50 | 73.12 | 82.81 | Ensemble |
| S9 | 40 | 53.75 | 46.87 | Decision Tree | 93.75 | 75.62 | 84.68 | SVM |
| S10 | 50.62 | 42.50 | 46.56 | Decision Tree | 89.37 | 83.12 | 86.25 | Ensemble |
| S11 | 40.62 | 54.37 | 47.50 | KNN | 83.75 | 89.37 | 86.56 | SVM |
| S12 | 56.25 | 53.12 | 54.68 | KNN | 90 | 68.12 | 79.06 | SVM |
| S13 | 48.12 | 56.25 | 52.18 | Decision Tree | 93.75 | 99.37 | 96.56 | SVM |
| S14 | 46.25 | 55.62 | 50.93 | KNN | 88.75 | 87.50 | 88.12 | SVM |
| S15 | 48.12 | 54.37 | 51.25 | KNN | 91.25 | 83.75 | 87.50 | SVM |
| Mean ± SD | 49.41 ± 7.69 | 50.41 ± 7.02 | 49.91 ± 2.94 | - | 90.45 ± 6.38 | 85.62 ± 8.46 | 87.90 ± 4.38 | - |

**S5 Table. Results of application of optimized machine learning approach for each subject.**
